# Biosafety and Biosecurity in Containment: A Regulatory Overview

**DOI:** 10.3389/fbioe.2020.00650

**Published:** 2020-06-30

**Authors:** Delphine S. A. Beeckman, Patrick Rüdelsheim

**Affiliations:** ^1^BASF Belgium Coordination Center CommV – Innovation Center Gent, Ghent, Belgium; ^2^PERSEUS bv, Sint-Martens-Latem, Belgium

**Keywords:** biosafety, biosecurity, biological material, biological agent, containment, regulations, (bio)risk assessment

## Abstract

When biosafety for contained use is addressed in international fora and discussions, often the topic is limited to working with genetically modified organisms (GMOs) in facilities such as laboratories, animal facilities, and greenhouses. However, the scope of biosafety in containment encompasses many other types of biological materials, such as human, animal and plant pathogens, nucleic acids, proteins, human samples, animals or plants, or by-products thereof, and overlaps often with the topic of biosecurity. This is also reflected in the regulations that apply for activities with biological materials in contained facilities. The common denominator of these regulations is the focus on protection of people and environment, while applying the key principles of risk assessment and risk management. This review provides an overview of regulatory frameworks for biosafety and biosecurity in containment around the globe, as well as points out overlap with other regulatory frameworks, such as the Nagoya Protocol, or Plant and Animal Health regulations.

## Introduction

“Biosafety” has multiple accepted definitions depending on the discipline involved (veterinary, food, medical, environmental, or space science), its linguistic roots or even the country in which it is used. Here are a few examples:

- “Safety with respect to the effects of biological research on humans and the environment” (Merriam-Webster, 2019).
- “(Laboratory) biosafety describes the containment principles, technologies, and practices that are implemented to prevent the unintentional exposure to pathogens and toxins, or their accidental release” (WHO, 2006).
- “Principles and practices for the prevention of unintentional release of or accidental exposure to biological agents and toxins” (OIE, 2017).
- “Practices and controls that reduce the risk of unintentional exposure or release of biological materials” (ISO, 2019).
- “The need to protect human health and the environment from the possible adverse effects of the products of modern biotechnology,” i.e., the concept of biosafety as described in the introduction of the Cartagena Protocol (SCBD, 2000).
- In terms of outer space, the concept of biosafety is referred to as “planetary protection”—“the practice of protecting solar system bodies (i.e., planets, moons, comets, and asteroids) from contamination by Earth life, and protecting Earth from possible life forms that may be returned from other solar system bodies” (NASA, 2019).

Also the term is sometimes used interchangeably with “biosecurity”, although this in itself has many different definitions:

- “Security from exposure to harmful biological agents; also: measures taken to ensure this security” (Merriam-Webster, 2019).
- “(Laboratory) biosecurity describes the protection, control, and accountability for Valuable Biological Materials agents and toxins within laboratories, in order to prevent their loss, theft, misuse, diversion of, unauthorized access, or intentional unauthorized release” (WHO, 2006).
- “A set of management and physical measures designed to reduce the risk of introduction, establishment and spread of animal diseases, infections or infestations to, from and within an animal population” (OIE, 2017).
- (Farm) biosecurity is a “set of measures designed to protect a property from the entry and spread of pests, diseases, and weeds” (AHA/PHA, 2019).
- “Encompasses all policy and regulatory frameworks (including instruments and activities) to manage risks associated with food and agriculture (including relevant environmental risks) including fisheries and forestry and constitutes three sectors (namely food safety, plant life and health, and animal life and health)” (FAO/IPPC, 2019b).
- “Practices and controls that reduce the risk of loss, theft, misuse, diversion of, or intentional unauthorized release of biological materials” (ISO, 2019).
- “The exclusion, eradication, or management of pests and diseases that pose a risk to the economy, environment, cultural and social values, including human health” (MPI, 2016).

Finally, some approaches refer to biorisk management as “coordinated activities to direct and control an organization with regard to biorisk”, i.e., “effect of uncertainty expressed by the combination of the consequences of an event (including changes in circumstances) and the associated ‘likelihood' of occurrence, where biological material is the source of harm” (ISO, 2019).

As a result of this diversity, “biosafety” and “biosecurity” are frequently used without any agreed definition or scope. The National Research Council (2009) summarizes the difference clearly: “Biosafety is about protecting people from bad ‘bugs'; biosecurity is about protecting ‘bugs' from bad people”.

For the purpose of this article, the following definitions relevant *for contained use* are used:

- **Biosafety**: Containment principles, technologies and practices that are implemented to prevent the unintentional exposure to biological material or their accidental release (adapted from WHO, 2006).
- **Biosecurity**: The protection, control, and accountability for biological agents and toxins within facilities in order to prevent their loss, theft, misuse, diversion, unauthorized access, or intentional unauthorized release (adapted from WHO, 2006).

With regard to “Containment,” the concept is generally accepted as “A set of measures including biological containment, practices, safety equipment, and facility safeguards that protect workers, the community and the environment from exposure to and/or unintentional escape of biological material” (adapted from WHO, 2004).

In this paper, we review a selection of objectives that drive the implementation of biosafety and biosecurity in contained environments and how these have been implemented in different parts of the world. Without advocating a specific approach, the review intends to highlight that different systems have been put in place to ensure safety when working with biological material, ranging from voluntary practices to legal requirements.

## Biosafety Objectives

### Protecting Workers and the Public Against Hazardous Biological Agents

Concrete references to biosafety practices in microbiology laboratories date from the time of Pasteur and Koch (period of the 1860's–1890's), when, following the first reports of disease in laboratory personnel, the need was identified to implement safety measures in response to potential risks associated with exposure to micro-organisms cultured in the lab. Being able to link certain diseases (e.g., anthrax, tuberculosis, and cholera) to their respective causative agents, Koch decided to handle them in a glazed tabletop box with two openings fitted with oilcloth sleeves. Although far from perfect, the idea of “bio-containment” was born (Berlinger, 2003).

Further research in the domain of laboratory acquired infections (LAIs) in microbiology laboratories contributed considerably to the adoption of protective measures against biological risks (Sulkin and Pike, 1949, 1951; Collins and Grange, 1990). These typically involved a combination of physical containment measures, working practices and personal protective equipment, focusing mainly on occupational safety. Simultaneously, also the US Biological Warfare (BW) program led to innovations in biosafety practices, which were shared at annual conferences starting from 1955 onwards. Although initially restricted to BW laboratories, in the sixties the audience was soon broadened to institutes and agencies involved in health and biomedical research, much to the benefit of their employees and public health (Barbeito and Kruse, 1997; Kruse and Barbeito, 1997a,b).

### Protecting Animal and Plant Health

With the development of global trade, the need to prevent and control the introduction and spread of pests of plants and plant products became more important. This led to the foundation of the International Plant Protection Convention (IPPC) in 1951, a multilateral treaty deposited with the Food and Agriculture Organization of the United Nations (FAO). The IPPC is the standard setting organization for the “Agreement on the Application of Sanitary and Phytosanitary Measures” (the SPS Agreement) of the World Trade Organization (WTO). Specific “International Standards for Phytosanitary Measures” (ISPMs) cover topics such as lists of quarantine organisms, pest risk analysis, or the design of plant quarantine stations, all of which are relevant when applying plant pests under containment in a laboratory or plant growing facility (FAO/IPPC, 2019a).

Similarly, to ensure safe global trade of animals and animal products while avoiding unnecessary obstructions to trade, the World Organization for Animal Health (OIE - Office International des Epizooties, est. 1924) is since 1998 the WTO reference organization for standards relating to animal health and zoonoses (WTO, 2019). The “Terrestrial Animal Health Code” and “Aquatic Animal Health Code” were developed with the aim of assuring the sanitary safety of international trade in terrestrial animals and aquatic animals, respectively, as well as their products. Traditionally addressing animal health and zoonoses only, these codes have been expanded to also cover animal welfare, animal production, and food safety in recent updates (OIE, 2019). As such, they provide concrete guidance for veterinary biosafety aspects of risk management and containment in veterinary research and diagnostic facilities.

Both plant protection and veterinary biosafety not only deal with the actual pathogens, but also define measures to control the vectors of either plant or animal/human diseases, such as arthropods or animal vectors.

### Dealing With Uncertainty/Protecting the Environment

Following the discovery of nucleic acids as the central molecules of heredity, the 1970s mark the emergence of a new discipline—molecular biology—with the first experiments with recombinant DNA and cloning being performed in the United States (Jackson et al., 1972). However, in parallel with the discovery of new techniques, questions quickly arose on possible risks associated with these types of experiments, especially because they were largely performed by biochemists less experienced in managing biological risks compared to microbiologists. Following discussions in 1973 (First Asilomar Conference, 1973 and Gordon Conference on Nucleic Acids, 1973), an appeal was made for a voluntary moratorium on experiments involving recombinant DNA until an international conference to assess the potential risks of such experiments was held (Berg et al., 1974). The Second Asilomar Conference (1975), bringing together scientists, legal experts, physicians and journalists adopted two basic principles:

- Containment should be an essential consideration in the experimental design;
- The effectiveness of the containment should match the estimated risk as closely as possible.

In addition, the conference also recommended biological and physical containment barriers as well as the adherence to good microbiological practices, and described a classification of experiments and corresponding containment levels.

One year later, the World Health Organization (WHO, 1976) launched the idea of applying the safety measures successfully implemented in microbiology to contain pathogenic organisms also for recombinant DNA experiments. In response, the National Institutes of Health (NIH) published the first “Guidelines for Research Involving Recombinant DNA Molecules” (NIH, 1976), enabling advances in life science, while promoting the safety of researchers, public and the environment. The “NIH guidelines”, revised in 1979, were used as a starting point for many regulations on contained use. Subsequently, some legal frameworks were established to formalize this for specific classes of organisms, referred to as Genetically Modified Organisms (GMOs). As technical progress is moving fast, uncertainty is often used to justify a precautionary approach, further requesting biosafety management for developments of genome editing and synthetic biology.

## Biosecurity Objectives

### Protection Against Loss, Theft, Misuse, Diversion, or Intentional Release

The WHO Biorisk Management Laboratory Biosecurity Guidance (WHO, 2006) introduced the concept of valuable biological materials (VBM). It is defined as “biological materials that require (according to their owners, users, custodians, caretakers, or regulators) administrative oversight, control, accountability, and specific protective and monitoring measures in laboratories to protect their economic and historical (archival) value, and/or the population from their potential to cause harm”. VBM may include pathogens and toxins, as well as non-pathogenic organisms, vaccine strains, foods, GMOs, cell components, genetic elements, and extraterrestrial samples. Next to possible theft, misuse, or intentional release of these VBM, there is also the concern that *bona fide* knowledge obtained from working with these materials in a research setting may at a later timepoint be misused to threaten public and animal health, food security, or the environment, also referred to as “dual use” or “dual use research of concern”. Hence, dual use considerations should be an essential part of a biosecurity program.

While laboratory biosafety and biosecurity manage different risks, “they share a common goal: keeping VBM safely and securely inside the areas where they are used and stored” (WHO, 2006).

### Preventing Development of Biological Weapons and Addressing Bioterrorism

Following the first World War, marked by the massive use of chemical weapons, several initiatives were undertaken to stop the chemicals arms race and restrict chemical warfare, albeit most of them were restricted to only a few countries (e.g., “Treaty of Versailles, 1919”), or failed to get ratified by all parties [e.g., “Washington Treaty (1922) in Relation to the Use of Submarines and Noxious Gases in Warfare” in 1922]. Negotiations were more successful in Geneva in 1925, with the signing of the “The Protocol for the Prohibition of the Use in War of Asphyxiating, Poisonous or other Gases, and of Bacteriological Methods of Warfare,” usually called the Geneva Protocol (1925). On proposal by the Polish representative, it was the first international agreement that included biological weapons as a separate arms category. However, only the use—and not the development or possession—of chemical and biological weapons was banned. Many signatories reserved the right to retaliate in-kind against states that violated the Protocol, making it *de facto* more of a “no-first-use” agreement. It took until 1972, with the “Convention on the Prohibition of the Development, Production and Stockpiling of Bacteriological (Biological) and Toxin Weapons and on Their Destruction” (commonly known as the “Biological Weapons Convention” or BWC), before also the development, production, storage, or otherwise acquiring or retaining biological agents and toxins, or related biological weapons or equipment, was prohibited. Exceptions are the application of such materials for prophylactic, protective, and other peaceful purposes (BWC, 1972). A group of 43 State Parties to the BWC has joined forces in the so-called “Australia Group”, an informal forum for countries to assist in the implementation of consistent export controls on goods that might contribute to the proliferation of biological or chemical weapons, thereby fulfilling their obligations to both the BWC and the Chemical Weapons Convention (AG, 2020).

## Addressing Biosafety and Biosecurity Objectives in Containment

While the objectives are clearly different, it is evident that biosafety and biosecurity are complementary disciplines that benefit from an aligned approach. It is therefore not surprising that biosafety and biosecurity in containment are often addressed together through a single biorisk management program, ensuring compliance with the requirements and good practices set out in both international guidance documents as well as in the different local legislative frameworks.

### International Framework and Guidance Documents

On the international level, different organizations and conventions with relevance for biosafety and biosecurity in containment are established, most of which derive from the United Nations (UN) or operate with it in close cooperation. These include, amongst others, the World Health Organization (WHO), the World Trade Organization (WTO), the World Organization for Animal Health (OIE), the Food and Agriculture Organization (FAO), the International Plant Protection Convention (IPPC), the Convention on Biological Diversity (CBD) and its associated Cartagena and Nagoya Protocols, and the Biological Weapons Convention (BWC), and their historical involvement is described in more detail elsewhere in this article.

These organizations and conventions provide governance on biosafety and biosecurity through a set of internationally accepted reference documents setting out objectives, principles, and requirements. Depending on the document, some of them have a legal basis while others are considered as best practices documents. A non-exhaustive list is given here:

- “WHO Biorisk Management: Laboratory Biosecurity Guidance” WHO/CDS/EPR/2006.6 (WHO, 2006).
- “WHO Laboratory Biosafety Manual: Third edition” WHO/CDS/CSR/LYO/2004.11 (WHO, 2004).
- “WHO International Health Regulations (2005): Third edition” (WHO, 2005) and the associated “Joint External Evaluation (JEE) tool” (WHO, 2016).
- “ISO 35001:2019: Biorisk management for laboratories and other related organizations” (ISO, 2019).
- “OIE Terrestrial Animal Health Code” (“Terrestrial Code”), 28th Ed., 2019.
- “OIE Manual of Diagnostic Tests and Vaccines for Terrestrial Animals” (“Terrestrial Manual”), 8th Ed., 2018.
- “OIE Aquatic Animal Health Code” (“Aquatic Code”), 22nd Ed., 2019.
- “OIE Manual of Diagnostic Tests for Aquatic Animals” (“Aquatic Manual”), 7th Ed., 2016.
- “IPPC Design and operation of post-entry quarantine stations for plants” (“ISPM 34”), 2016.
- “NIH Guidelines for Research Involving Recombinant or Synthetic Nucleic Acid Molecules” (“NIH Guidelines”), April 2019.
- Biosafety in Microbiological and Biomedical Laboratories” (“BMBL”), 5th Ed., 2009.
- “CDC Guidelines for Safe Work Practices in Human and Animal Medical Diagnostic Laboratories,” 2012.
- “Canadian Biosafety Standard” (CBS), 2nd Ed., 2015.
- “Canadian Biosafety Handbook” (CBH), 2nd Ed., 2015.

Many of these internationally accepted reference documents share the same basic principles: (1) a classification system for the biological agents or biological materials in so-called risk groups, often divided into four classes going from 1 (low) to 4 (high); (2) the understanding that increasing occupational and environmental risks require more stringent containment measures to work with that material, which is translated in a requirement for both risk assessment and risk management that is tailored to the activities performed with the biological materials, and (3) the description of containment measures, either result-oriented or more prescriptive as true containment or biosafety levels (Table 1).

**Table 1:** Overview of principles shared among internationally accepted reference documents for biosafety in containment.

**Topic**	**WHO LBM**	**35001**	**BMBL**	**NIH G**	**CDC G**	**CBS + CBH**
Risk groups	X	-	X	X	(X)	X
Activity based risk management	X	X	X	X	X	X
Containment measures – prescriptive	X	-	X	X	X	X
Containment measures – result oriented	-	X	-	-	X	X

*Legend: WHO LBM, WHO Laboratory Biosafety Manual; 35001, ISO 35001:2019 Biorisk management for laboratories and other related organizations; BMBL, Biosafety in Microbiological and Biomedical Laboratories 5th ed.; NIH G, NIH Guidelines for Research Involving Recombinant or Synthetic Nucleic Acid Molecules; CDC G, CDC Guidelines for Safe Work Practices in Human and Animal Medical Diagnostic Laboratories; CBS, Canadian Biosafety Standard (CBS), 2nd Ed.; CBH, Canadian Biosafety Handbook (CBH), 2nd Ed*.

Some of these reference documents have also served as the foundation for the development of national biosafety and biosecurity legislation, regulations and policies, either by including and refining the concepts mentioned in these documents or including the compliance with these documents as a requirement in the legislation.

### Examples of Country- or Region-Specific Legislation

Due to the multiple objectives envisaged by biosafety and biosecurity (*vide infra*), regulatory requirements are most often part of legislation that is focusing on topics such as Worker Protection, Activities with Genetically Modified Organisms (GMO), Activities with Pathogens (human, animal, plant, quarantine), Waste or Biosecurity.

We have compiled the specific references to biosafety and biosecurity aspects in these themes for key countries in Appendix 1 – Part A. These overviews were prepared for Australia, Brazil, Canada, the European Union, Singapore and the United States of America, and they reflect the regulatory status at the time of compilation (period July – Dec 2019) as examples of different approaches (Readers are advised to consult the local regulations to have access to the updated and most recent information).

## Overlaps With Other Regulatory Frameworks That Have Provisions on Handling Biological Materials

Both on the international and the regional or local level, additional provisions for handling of biological materials are imbedded in diverse regulatory texts, several of which on first sight would not be immediately recognized as being relevant for biosafety and biosecurity in containment. Many of them are related to the topic of transboundary movement, traceability, transport and occupational hygiene, and their link to biosafety and biosecurity for contained use is explained here further for some concrete examples.

### Cartagena Protocol

The “Cartagena Protocol on Biosafety to the Convention on Biological Diversity” (SCBD, 2000) describes in its Article 18 that “LMOs [Living Modified Organisms] that are subject to intentional transboundary movement within the scope of the Protocol are [to be] handled, packaged, and transported under conditions of safety, taking into consideration relevant international rules and standards”, thus clearly referring to existing rules and requirements for maintaining containment during transport. Specifically for LMOs that are destined for contained use it is stipulated that they should be clearly identified as LMOs, a requirement which is common to many GMO specific regulations in different countries, and shipment documentation should provide instructions for the safe handling, storage, transport and use, thereby ensuring containment. In addition, by means of Article 15 “Risk Assessment” (including Annex III) and Article 16 “Risk Management”, the Cartagena Protocol is aligned with the concepts described in different internationally accepted reference documents for biosafety in containment (see International Framework and Guidance Documents).

### Nagoya Protocol

The “Nagoya Protocol on Access to Genetic Resources and the Fair and Equitable Sharing of Benefits Arising from their Utilization to the Convention on Biological Diversity” (SCBD, 2011) states that when benefits (either monetary or non-monetary) are arising from the utilization of genetic resources (e.g., in research) as well as during subsequent commercialization, that these benefits “shall be shared in a fair and equitable way with the Party providing such resources that is the country of origin of such resources or a Party that has acquired the genetic resources in accordance with the Convention”. Although in principle not related to biosafety, the Nagoya Protocol implies that full traceability on when and where a certain genetic resource (i.e., biological material, or in some case arguably even digital sequence information) was first accessed, as well as how it was subsequently used, is maintained. Clearly specifying the identity of biological material and ensuring traceability is also a key element of biorisk management. Typically, this traceability involves both biological traceability (from one generation to the next) as well as physical traceability (when shipped from one location to another) and recording requires the information to be updated in inventories, which are also a prerequisite to identify the hazards associated with an activity. In addition, appropriate inventories for regulated materials are often a legal requirement from a biosafety contained use perspective in certain countries or regions.

### Plant and Animal Health

In the section on “Protecting Animal and Plant Health” the efforts from the International Plant Protection Convention (IPPC) and the World Organization for Animal Health (OIE) in safeguarding containment when handling plant and animal pathogens, respectively, were highlighted.

However, many of the standards developed by these two organizations deal with topics such as import and export as well as traceability. This is especially important in case of newly emerging infections with the potential of world-wide epidemics. Checking the sanitary status of plant materials and animals prior to import or export reduces the risk of spreading diseases, while the recording of movements is imperative to allow for a quick and targeted response in case it does go wrong.

### Occupational Hygiene

Occupational Hygiene, as defined by the International Occupational Hygiene Association (IOHA, 2020) is “the discipline of anticipating, recognizing, evaluating, and controlling health hazards in the working environment with the objective of protecting worker health and well-being and safeguarding the community at large”. Also known under the term of Industrial Hygiene, it is typically part of an Occupational Safety and Health program, where it focuses on chemical, physical and biological agents in the workplace possibly causing illness or discomfort, and aims to avoid health effects through risk assessment and management. Although occupational hygiene and biosafety go hand in hand in terms of both intended and unintended exposure to biological agents, there is a clear difference in scope, being the general workplace as a whole vs. specific activities with biological materials, respectively. A clear example in this respect in the prevention against Legionnaires disease (*Legionella*), which is a typical workplace biological exposure monitored and managed by occupational hygiene, and generally not in scope of biosafety.

### Transport Regulations

The UN Model Regulations from the UN Economic and Social Council's Committee of Experts (UNECE) on the Transport of Dangerous Goods describe the recommendations for transport of dangerous goods to safeguard workers' health and safety, property, or environment protection during all modes of transport. These dangerous goods are divided in 9 classes, one of which is devoted to toxic and infectious substances (Class 6), while GMOs are classified as miscellaneous dangerous substances (Class 9). For each class of dangerous goods, the UN Model Regulations cover aspects such as general packing requirements, labeling, and transport documents. Although they are only recommendations, they serve as the basis for national and international transport regulations, and as such, contribute to worldwide harmonization in this field (UNECE, 2020). For infectious materials, triple packaging (consisting out of leakproof primary and secondary receptacles) is the rule, to ensure containment of the biological materials during transport and in the event of accidents or incidents. As such, when biological materials are brought outside of containment for transport, appropriate packaging ensures protection from unintentional exposure or accidental release.

Specific references to the national or regional legislation for the above-mentioned biosafety-related topics are given in Appendix 1 – Part B (Readers are advised to consult the local regulations to have access to the updated and most recent information).

## Conclusion

Although biosafety and biosecurity serve different objectives, they are often addressed together, especially in a contained use setting. This discipline has a long-standing history, predating GMO-focused biosafety approaches, and continues to evolve as new insights and new techniques become available. The risk assessment and management practices are embedded in a vast and robust framework of international, regional and national regulations and guidance dealing with handling, storage, containment measures, waste management, transport, packaging, and labeling of biological organisms under contained use, including GMOs, thereby ensuring the protection of human, animal, and plant health as well as the environment. Local (national, regional) legislation may be influenced by policy priorities, leading to significant differences in the administrative aspects of how biosafety is regulated, however, the main principles and practices are shared worldwide. And, as experience has shown, when new developments in biotechnology, microbiology, and synthetic biology emerge, the existing frameworks and practices can be applied and tailored when needed.

## Author Contributions

DB and PR co-developed the concept of the manuscript. DB wrote the first draft of the manuscript. All authors contributed to manuscript revision, read and approved the submitted version.

## Conflict of Interest

The authors declare that the research was conducted in the absence of any commercial or financial relationships that could be construed as a potential conflict of interest.
