# Supplementary material for: Biosafety and Biosecurity in Containment: A Regulatory Overview
Source: Front Bioeng Biotechnol. 2020 Jun 30;8:650. doi: 10.3389/fbioe.2020.00650 (PMC7348994; doi:10.3389/fbioe.2020.00650)
Supplement: Supplementary file 1 [file Table_1.docx]

**Appendix 1**

Part A: Legislative references to biosafety and biosecurity objectives covered by legislation that is focusing on topics such as Worker Protection, Activities with Genetically Modified Organisms (GMO), Activities with Pathogens (human, animal, plant, quarantine), Waste or Biosecurity.

| **Subject** | EU | USA | Canada | Australia | Singapore | Brazil |
| --- | --- | --- | --- | --- | --- | --- |
| Worker Protection | **1.1** | **2.1** | **3.1** | **4.1** | **5.1** | **6.1** |
| GMO Contained Use and Deliberate Release | **1.2** | **2.2** | **3.2** | **4.2** | **5.2** | **6.2** |
| Pathogen Work – Plants | **1.3** | **2.3** | **3.3** | **4.3** | **5.3^b^** | **6.3** |
| Pathogen Work – Animals | **1.4** | **2.4** | **3.4** | **4.4^a^** | **5.4** | **6.4^b^** |
| Waste | **1.5** | **2.5** | **3.5^a^** | **4.5^a^** | **5.5** | **6.5^b^** |
| Dual-use Items / DURC / Biosecurity | **1.6** | **2.6** | **3.6** | **4.6** | **5.6** | **6.6^b^** |

**X.X** = Link to legislation identified for this topic

**^a^** There is information on this topic, but no dedicated legislation could be identified

**^b^** No legislation could be identified for this topic

Part B: Legislative references to biosafety and biosecurity related topics such as transboundary movement, traceability, transport and occupational hygiene.

| **Subject** | EU | USA | Canada | Australia | Singapore | Brazil |
| --- | --- | --- | --- | --- | --- | --- |
| GMO Transboundary Movement – Cartagena Protocol Implementation | **1.7** | **2.7^a^** | **3.7^a^** | **4.7^a^** | **5.7^a^** | **6.7** |
| Access and Benefit Sharing of Genetic resources – Nagoya Protocol Implementation | **1.8** | **2.8^a^** | **3.8^a^** | **4.8^a^** | **5.8^a^** | **6.8^b^** |
| Plant Health –  Import/Export Regulations, Traceability | **1.9** | **2.9** | **3.9** | **4.9** | **5.9** | **6.9** |
| Animal health –  Notifiable Diseases, Emergency Measures | **1.10** | **2.10** | **3.10** | **4.10** | **5.10** | **6.10^b^** |
| Occupational Hygiene (e.g. *Legionella*) | **1.11** | **2.11** | **3.11** | **4.11^a^** | **5.11^b^** | **6.11^a^** |
| Transport | **1.12** | **2.12** | **3.12** | **4.12^a^** | **5.12^a^** | **6.12^a^** |

**X.X** = Link to legislation identified for this topic

**^a^** There is information on this topic, but no dedicated legislation could be identified

**^b^** No legislation could be identified for this topic

# European Union Status: Sep ’19 [(home)](#_top)

- 1. Worker Protection [(home)](#_top)

Directive 2000/54/EC of the European Parliament and of the Council of 18 September 2000 on the protection of workers from risks related to exposure to biological agents at work.

<http://data.europa.eu/eli/dir/2000/54/oj>

- 1. GMO Contained Use and Deliberate Release [(home)](#_top)

Directive 2009/41/EC of the European Parliament and of the Council of 6 May 2009 on the contained use of genetically modified micro-organisms. <http://data.europa.eu/eli/dir/2009/41/oj>

Directive 2001/18/EC of the European Parliament and of the Council of 12 March 2001 on the deliberate release into the environment of genetically modified organisms. <http://data.europa.eu/eli/dir/2001/18/oj>

- 1. Pathogen Work – Plants [(home)](#_top)

**Before Dec 14, 2019:**

Council Directive 2000/29/EC of 8 May 2000 on protective measures against the introduction into the Community of organisms harmful to plants or plant products and against their spread within the Community. <http://data.europa.eu/eli/dir/2000/29/oj>

Commission Directive 2008/61/EC of 17 June 2008 establishing the conditions under which certain harmful organisms, plants, plant products and other objects listed in Annexes I to V to Council Directive 2000/29/EC may be introduced into or moved within the Community or certain protected zones thereof, for trial or scientific purposes and for work on varietal selections. <http://data.europa.eu/eli/dir/2008/61/oj>

**As of Dec 14, 2019:**

Regulation (EU) 2016/2031 of the European Parliament of the Council of 26 October 2016 on protective measures against pests of plants (“Plant Health Law”) and associated implementing and delegated acts. <http://data.europa.eu/eli/reg/2016/2031/oj>

Commission Delegated Regulation (EU) 2019/829 of 14 March 2019 supplementing Regulation (EU) 2016/2031 of the European Parliament and of the Council on protective measures against pests of plants, authorizing Member States to provide for temporary derogations in view of official testing, scientific or educational purposes, trials, varietal selections, or breeding. <http://data.europa.eu/eli/reg_del/2019/829/oj>

- 1. Pathogen Work – Animals [(home)](#_top)

**Before April 21, 2021:**

Directive 2010/63/EU of the European Parliament and of the Council of 22 September 2010 on the protection of animals used for scientific purposes. <http://data.europa.eu/eli/dir/2010/63/oj>

The European legislation is extremely complex with currently very many different acts that cover individual or several quarantine animal pests and diseases (as listed by the OIE – World Organization for Animal health). These requirements will be combined into a single Animal Health Framework, applicable as of April 21, 2021.

**As of April 21, 2021:**

[Regulation (EU) 2016/429 of the European Parliament and of the Council of 9 March 2016 on transmissible animal diseases and amending and repealing certain acts in the area of animal health (“Animal Health Law”)](https://eur-lex.europa.eu/legal-content/EN/AUTO/?uri=uriserv:OJ.L_.2016.084.01.0001.01.ENG&toc=OJ:L:2016:084:TOC) and associated implementing and delegated acts. <http://data.europa.eu/eli/reg/2016/429/oj>

- 1. Waste [(home)](#_top)

Directive 2008/98/EC of the European Parliament and of the Council of 19 November 2008 on waste (“Waste Framework Directive”). <http://data.europa.eu/eli/dir/2008/98/oj>

Commission Decision 2000/532/EC of 3 May 2000 replacing Decision 94/3/EC establishing a list of wastes pursuant to Article 1(a) of Council Directive 75/442/EEC on waste and Council Decision 94/904/EC establishing a list of hazardous waste pursuant to Article 1(4) of Council Directive 91/689/EEC on hazardous waste. <http://data.europa.eu/eli/dec/2000/532/oj>

Regulation (EC) No 1069/2009 of the European Parliament and of the Council of 21 October 2009 laying down health rules regarding animal by-products and derived products not intended for human consumption and repealing Regulation (EC) No 1774/2002 (“Animal by-products Regulation”). <http://data.europa.eu/eli/reg/2009/1069/oj>

Regulation (EC) No 1013/2006 of the European Parliament and of the Council of 14 June 2006 on shipments of waste. <http://data.europa.eu/eli/reg/2006/1013/oj>

- 1. Dual-Use Items / DURC / Biosecurity [(home)](#_top)

Council Regulation (EC) No 428/2009 of 5 May 2009 setting up a Community regime for the control of exports, transfer, brokering and transit of dual-use items. <http://data.europa.eu/eli/reg/2009/428/oj>

- 1. GMO Transboundary Movement – Cartagena Protocol Implementation [(home)](#_top)

Regulation (EC) No 1946/2003 of the European Parliament and of the Council of 15 July 2003 on transboundary movements of genetically modified organisms. <http://data.europa.eu/eli/reg/2003/1946/oj>

Regulation (EC) No 882/2004 of the European Parliament and of the Council of 29 April 2004 on official controls performed to ensure the verification of compliance with feed and food law, animal health and animal welfare rules. <http://data.europa.eu/eli/reg/2004/882/oj>

**As of Dec 14, 2019:**

Regulation (EU) 2017/625 of the European Parliament and of the Council of 15 March 2017 on official controls and other official activities performed to ensure the application of food and feed law, rules on animal health and welfare, plant health and plant protection products (“Official Controls Regulation”). <http://data.europa.eu/eli/reg/2017/625/oj>

- 1. Access and Benefit Sharing of Genetic Resources – Nagoya Protocol Implementation [(home)](#_top)

Regulation (EU) No 511/2014 of the European Parliament and of the Council of 16 April 2014 on compliance measures for users from the Nagoya Protocol on Access to Genetic Resources and the Fair and Equitable Sharing of Benefits Arising from their Utilization in the Union. <http://data.europa.eu/eli/reg/2014/511/oj>

Commission Implementing Regulation (EU) 2015/1866 of 13 October 2015 laying down detailed rules for the implementation of Regulation (EU) No 511/2014 of the European Parliament and of the Council as regards the register of collections, monitoring user compliance and best practices. <http://data.europa.eu/eli/reg_impl/2015/1866/oj>

- 1. Plant Health – Import/Export Regulations, Traceability [(home)](#_top)

**As of Dec 14, 2019:**

Regulation (EU) 2016/2031 of the European Parliament of the Council of 26 October 2016 on protective measures against pests of plants (“Plant Health Law”) and associated implementing and delegated acts. <http://data.europa.eu/eli/reg/2016/2031/oj>

Regulation (EU) 2017/625 of the European Parliament and of the Council of 15 March 2017 on official controls and other official activities performed to ensure the application of food and feed law, rules on animal health and welfare, plant health and plant protection products (“Official Controls Regulation”). <http://data.europa.eu/eli/reg/2017/625/oj>

- 1. Animal health – Notifiable Diseases, Emergency Measures [(home)](#_top)

The current regulatory landscape in the European Union regarding Animal Health is fragmented, as most legislation is focusing on specific diseases or (groups of) animals. The existing series of linked and interrelated legal acts will be streamlined into single law (‘Animal Health Law’).

**As of Dec 14, 2019:**

Regulation (EU) 2017/625 of the European Parliament and of the Council of 15 March 2017 on official controls and other official activities performed to ensure the application of food and feed law, rules on animal health and welfare, plant health and plant protection products (“Official Controls Regulation”). <http://data.europa.eu/eli/reg/2017/625/oj>

**As of April 21, 2021:**

[Regulation (EU) 2016/429 of the European Parliament and of the Council of 9 March 2016 on transmissible animal diseases and amending and repealing certain acts in the area of animal health (‘Animal Health Law’)](https://eur-lex.europa.eu/legal-content/EN/AUTO/?uri=uriserv:OJ.L_.2016.084.01.0001.01.ENG&toc=OJ:L:2016:084:TOC) and associated implementing and delegated acts. <http://data.europa.eu/eli/reg/2016/429/oj>

- 1. Occupational Hygiene (*e.g.* *Legionella*) [(home)](#_top)

Council Directive 89/391/EEC of 12 June 1989 on the introduction of measures to encourage improvements in the safety and health of workers at work. <http://data.europa.eu/eli/dir/1989/391/oj>

Directive 2000/54/EC of the European Parliament and of the Council of 18 September 2000 on the protection of workers from risks related to exposure to biological agents at work. <http://data.europa.eu/eli/dir/2000/54/oj>

- 1. Transport [(home)](#_top)

Within EU:

Directive 2008/68/EC of the European Parliament and of the Council of 24 September 2008 on the inland transport of dangerous goods. <http://data.europa.eu/eli/dir/2008/68/oj>

Worldwide:

ADR (road)

IATA (air)

IMDG (sea)

# United States of America Status: Aug ’19 [(home)](#_top)

- 1. Worker Protection [(home)](#_top)

Occupational Safety and Health Standards - 29 CFR 1910

<https://www.osha.gov/laws-regs/regulations/standardnumber/1910>

More specifically: OSHA Bloodborne Pathogens (BBP) 29 CFR 1910.1030

<https://www.osha.gov/pls/oshaweb/owadisp.show_document?p_id=10051&p_table=STANDARDS>

General information is available on <https://www.osha.gov/>

- 1. GMO Contained Use and Deliberate Release [(home)](#_top)

GMOs are regulated under the Coordinated Framework for Regulation of Biotechnology, pursuant to health, safety, and environmental legislation governing conventional products:

- Plant Protection Act (PPA) and Animal Health Protection Act (AHPA): Plant GMOs, GM animals and insects that pose a risk to animal health, regulated by the US Department of Agriculture’s Animal and Plant Health Inspection Service (USDA APHIS) (imports, interstate movements, and environmental releases). <https://www.aphis.usda.gov/aphis/ourfocus/biotechnology/SA_Program_Overview>

<https://www.aphis.usda.gov/animal_health/downloads/framework-ee-ahpa.pdf>

- - GE animals: 9 CFR Part 93 (import) and 9 CFR Part 71 (interstate movement)
  - GE Insects: 9 CFR Part 122
  - GE plants: 7 CFR Part 340
- Federal Food, Drug, and Cosmetic Act (FFDCA – 21 USC 9) and the Public Health Service Act (PHSA): GMOs in food, drugs (incl. GM animals), and biological products, regulated by the Food and Drug Administration (FDA). <https://www.fda.gov/safety/fdas-regulation-plant-and-animal-biotechnology-products>
  - Under FFDCA: 40 CFR Part 152; 40 CFR Part 174; 40 CFR Part 172
  - 21 CFR Chapter I (FDA)
- Federal Insecticide, Fungicide and Rodenticide Act (FIFRA - 7 USC 6) and the Toxic Substances Control Act (TSCA – 15 USC 53): GMO pesticides and microorganisms, regulated by the Environmental Protection Agency (EPA). <https://www.epa.gov/regulation-biotechnology-under-tsca-and-fifra/>
  - Under FIFRA: 40 CFR Part 152; 40 CFR 174; 40 CFR Part 172
  - TSCA: 40 CFR Part 700-799, especially 40 CFR Part 725
- Bioterrorism Agents Regulations:
  - 9 CFR Part 121; 7 CFR Part 331, 42 CFR Part 73

The form of regulation varies depending on the type of GMO involved. Depending on its characteristics, a product may be subject to the jurisdiction of one or more of these agencies. There is no regulation specifically on contained use of GMOs within laboratories and greenhouses, but import is regulated, often setting specific conditions for handling of the material, also in contained use (Note: contained use as defined in 40 CFR § 725.234 and § 725.235 “Activities conducted inside a structure”).

More information is available at:

- the Unified Website for Biotechnology Regulation: <https://usbiotechnologyregulation.mrp.usda.gov/biotechnologygov/home/>
- <https://www.loc.gov/law/help/restrictions-on-gmos/usa.php#Research>
- <https://www.aphis.usda.gov/aphis/ourfocus/biotechnology/sa_regulations/ct_biotech_laws_and_regs_framework>

In addition, when receiving federal funding for activities with GMOs, one needs to comply with the NIH Guidelines for Research Involving Recombinant or Synthetic Nucleic Acid Molecules (“NIH Guidelines”).

<https://osp.od.nih.gov/biotechnology/nih-guidelines/>

- 1. Pathogen Work – Plants [(home)](#_top)

Plant Protection Act (PPA) – 7 USC 104

<https://www.law.cornell.edu/uscode/text/7/chapter-104>

U.S. Regulated Plant Pest List

<https://www.aphis.usda.gov/aphis/ourfocus/planthealth/import-information/rppl>

- 1. Pathogen Work – Animals [(home)](#_top)

Animal Health Protection Act (APHA) – 7 USC 109

<https://www.law.cornell.edu/uscode/text/7/chapter-109>

- 1. Waste [(home)](#_top)

Waste disposal is state specific in the U.S., but the overarching regulation is the Resource Conservation and Recovery Act (RCRA) setting the framework for the proper management of hazardous and non-hazardous solid waste – 42 USC 82

<https://uscode.house.gov/view.xhtml?path=/prelim@title42/chapter82&edition=prelim>

More information is available at:

<https://www.epa.gov/regulatory-information-topic/regulatory-information-topic-waste>

Specific information on medical waste is available at:

<https://www.epa.gov/rcra/medical-waste>

- 1. Dual-Use Items / DURC / Biosecurity [(home)](#_top)

Several acts and policies are available – a non-exhaustive list is given below:

Select Agents and Toxins:

- Agriculture – Possession, Use, and Transfer of Select Agents and Toxins - 7 CFR 331. <https://www.law.cornell.edu/cfr/text/7/part-331>
- Animals and Animal Products - Possession, Use, and Transfer of Select Agents and Toxins – 9 CFR 121. <https://www.selectagents.gov/Regulations.html>
- Public Health – Select Agents and Toxins - 42 CFR 73. <https://www.law.cornell.edu/cfr/text/42/part-73>
- Foreign Relations – United States Munitions List – Category XIV Toxicological Agents, Including Chemical Agents, Biological Agents and Associated Equipment – 7 CFR 121 Section 121 Category XIV(b). <https://www.law.cornell.edu/cfr/text/22/121.1>
- Per Regulatory Agency
  - APHIS: 7 CFR 331.3(b), 9 CFR 121.3(b), and 9 CFR 121.4(b);
  - CDC: 42 CFR 73.3(b) and 42 CFR 73.4(b).
  - International Traffic in Arms Regulations (ITAR): 22 CFR part 121, Category XIV(b), for modified biological agents and biologically derived substances that are “subject to the ITAR”.
- Dedicated website on the topic: <https://www.selectagents.gov/>

Commerce Control List:

- Commerce Control List, Category 1 - 15 CFR Part 774 <https://www.law.cornell.edu/cfr/text/15/part-774>

Dual Use Research of Concern:

- Pursuant to Executive Order 13546 (*Optimizing the Security of Biological Select Agents and Toxins in the United States)* the U.S. Government issued two policies to address risks associated with DURC. <http://edocket.access.gpo.gov/2010/pdf/2010-16864.pdf>
  - *United States Government Policy for Oversight of Life Sciences Dual Use Research of Concern* (DURC Policy, March 2012)

<https://www.phe.gov/s3/dualuse/Documents/us-policy-durc-032812.pdf>

- - *United States Government Policy for Institutional Oversight of Dual Use Research of Concern*

<https://www.phe.gov/s3/dualuse/Documents/durc-policy.pdf>

- More information is available on the EPA website: <https://www.epa.gov/research/policy-and-procedures-managing-dual-use-research-concern>
- Framework for Guiding Funding Decisions about Proposed Research Involving Enhanced Potential Pandemic Pathogens: <https://www.phe.gov/s3/dualuse/Documents/P3CO.pdf>

Other:

- Public Health Security and Bioterrorism Preparedness and Response Act of 2002 <https://www.law.cornell.edu/topn/public_health_security_and_bioterrorism_preparedness_and_response_act_of_2002>
- 7 USC 110 – Enhancing Controls in Dangerous Biological Agents and Toxins <https://www.law.cornell.edu/uscode/text/7/chapter-110>

- 1. GMO Transboundary Movement – Cartagena Protocol Implementation [(home)](#_top)

The US is not a party to the Cartagena Protocol: <https://www.cbd.int/information/parties.shtml#tab=1>

- 1. Access and Benefit Sharing of Genetic Resources – Nagoya Protocol [(home)](#_top)

The US is not a party to the Nagoya Protocol: <https://www.cbd.int/information/parties.shtml#tab=2>

- 1. Plant Health – Import/Export regulations, Traceability [(home)](#_top)

Plant Protection Act (PPA) – 7 USC 104

<https://uscode.house.gov/view.xhtml?path=/prelim@title7/chapter104&edition=prelim>

U.S. Regulated Plant Pest List

<https://www.aphis.usda.gov/aphis/ourfocus/planthealth/import-information/rppl>

APHIS dedicated website on the topic:

<https://www.aphis.usda.gov/aphis/ourfocus/planthealth>

- 1. Animal Health – Notifiable Diseases, Emergency Measures [(home)](#_top)

Animal Health Protection Act (AHPA) – 7 USC 109

<https://www.govinfo.gov/app/details/USCODE-2011-title7/USCODE-2011-title7-chap109/context>

APHIS dedicated website on the topic:

<https://www.aphis.usda.gov/aphis/ourfocus/animalhealth>

- 1. Occupational Hygiene (*e.g. Legionella*) [(home)](#_top)

Occupational Safety and Health Act – 29 US Code 15 <https://www.govinfo.gov/app/details/USCODE-2011-title29/USCODE-2011-title29-chap15/context>

General information is available at:

<https://www.osha.gov/law-regs.html>

<https://www.cdc.gov/niosh/>

Specifically for Legionella: <https://www.osha.gov/SLTC/legionnairesdisease/index.html>

- 1. Transport [(home)](#_top)

Within USA

Transportation – Hazardous Materials Regulations - 49 CFR Subtitle B Chapter I Subchapter C

<https://www.law.cornell.edu/cfr/text/49/subtitle-B/chapter-I/subchapter-C>

Worldwide:

ADR (road)

IATA (air)

IMDG (sea)

# Canada Status: Aug ’19 [(home)](#_top)

- 1. Worker Protection [(home)](#_top)

Canada Labour Code (R.S.C., 1985, c. L-2)

<https://laws-lois.justice.gc.ca/eng/acts/L-2/>

- PART II - Occupational Health and Safety

<https://laws-lois.justice.gc.ca/eng/acts/L-2/page-23.html#h-341197>

Canada Occupational Health and Safety Regulations (SOR/86-304)

<https://laws-lois.justice.gc.ca/eng/regulations/SOR-86-304/>

- PART X - Hazardous Substances

[https://laws-lois.justice.gc.ca/eng/regulations/SOR-86-304/page-23.html#h-894311](https://laws-lois.justice.gc.ca/eng/regulations/SOR-86-304/page-23.html#h-894311 )

- PART XII - Protection Equipment and Other Preventive Measures

<https://laws-lois.justice.gc.ca/eng/regulations/SOR-86-304/page-33.html#h-895051>

- PART XV - Hazardous Occurrence Investigation, Recording and Reporting

[https://laws-lois.justice.gc.ca/eng/regulations/SOR-86-304/page-41.html#h-895604](https://laws-lois.justice.gc.ca/eng/regulations/SOR-86-304/page-41.html#h-895604 )

- PART XVII - Safe Occupancy of the Work Place

<https://laws-lois.justice.gc.ca/eng/regulations/SOR-86-304/page-50.html#h-895931>

- PART XIX - Hazard Prevention Program

<https://laws-lois.justice.gc.ca/eng/regulations/SOR-86-304/page-57.html#h-896504>

Human Pathogen and Toxins Act (S.C. 2009, c. 24)

<https://laws.justice.gc.ca/eng/acts/H-5.67/>

Human Pathogens and Toxins Regulations (SOR/2015-44)

<https://laws.justice.gc.ca/eng/regulations/SOR-2015-44/>

Canada's national resource for the advancement of workplace health and safety: <https://www.ccohs.ca/>

- 1. GMO Contained Use and Deliberate Release [(home)](#_top)

Living modified organisms are regulated under:

- For living modified plants
  - The Plant Protection Act (S.C. 1990, c. 22)

<https://laws-lois.justice.gc.ca/eng/acts/P-14.8/>

- - The Seeds Act (R.S.C., 1985, c. S-8)

<https://laws-lois.justice.gc.ca/eng/acts/S-8/>

- For living modified animals
  - The Canadian Environmental Protection Act 1999 (S.C. 1999, c. 33), Part 6 – Animate Products of Biotechnology

<https://laws-lois.justice.gc.ca/eng/acts/C-15.31/page-15.html#h-64413>

- For aquatic organisms
  - The Fisheries Act (R.S.C., 1985, c. F-14)

<https://laws-lois.justice.gc.ca/eng/acts/F-14/>

- For veterinary biologics
  - The Health of Animals Act (S.C. 1990, c. 21)

<https://laws-lois.justice.gc.ca/eng/acts/H-3.3/>

- New Substances Notification Regulations (Organisms) or NSNR(O) (SOR/2005-248). <https://laws-lois.justice.gc.ca/eng/regulations/SOR-2005-248/>
  - To avoid regulatory duplication, those organisms regulated under the *Seeds Act, Feeds Act, Fertilizers Act* (all administered by the CFIA), and HAA (with respect to veterinary biologics, administered by the CFIA), and the Pest Control Products Act (administered by the Pest Management Regulatory Agency) are exempt from the NSNR(O) for products or activities already covered by the legislation.
  - The NSNR(O) does not apply to a microorganism that is imported for use that is regulated under other acts or regulations (e.g., HPTA, HAA).
  1. Pathogen Work – Plants [(home)](#_top)

Plant Protection Act (S.C. 1990, c. 22).

<https://laws-lois.justice.gc.ca/eng/acts/P-14.8/>

Plant Protection Regulations (SOR/95-212).

<https://laws-lois.justice.gc.ca/eng/regulations/SOR-95-212/>

- 1. Pathogen Work – Animals [(home)](#_top)

Health of Animals Act (S.C. 1990, c. 21)

<https://laws-lois.justice.gc.ca/eng/acts/H-3.3/>

Health of Animals Regulations (C.R.C., c. 296)

<https://laws-lois.justice.gc.ca/eng/regulations/C.R.C.,_c._296/>

Human Pathogen and Toxins Act (S.C. 2009, c. 24)

<https://laws.justice.gc.ca/eng/acts/H-5.67/>

Human Pathogens and Toxins Regulations (SOR/2015-44)

<https://laws.justice.gc.ca/eng/regulations/SOR-2015-44/>

- 1. Waste [(home)](#_top)

The Canadian Council of Ministers of the Environment (CCME) published the document “Guidelines for the Management of Biomedical Waste in Canada” (1992) as a basis for development of local regulations. <https://www.ccme.ca/files/Resources/waste/hazardous/pn_1060_e.pdf>

- 1. Dual-Use Items / DURC / Biosecurity [(home)](#_top)

Dual-use pathogens and toxins, goods, and technology are regulated under

- For human pathogens, including zoonotic pathogens, or toxins
  - The Human Pathogen and Toxins Act (S.C. 2009, c. 24)

<https://laws.justice.gc.ca/eng/acts/H-5.67/>

- - Human Pathogens and Toxins Regulations (SOR/2015-44)

<https://laws.justice.gc.ca/eng/regulations/SOR-2015-44/>

- - Guidance document for research settings:

Plan for Administrative Oversight for Pathogens and Toxins in a Research Setting - Required Elements and Guidance

<https://www.canada.ca/content/dam/phac-aspc/documents/services/laboratory-biosafety-biosecurity/licensing-program/plan-administrative-oversight-pathogens-toxins-a-research-setting-required-elements-guidance/admin_oversight-surveillance_admin-eng.pdf>

- For animal pathogens
  - The Health of Animals Act (S.C. 1990, c. 21)

<https://laws-lois.justice.gc.ca/eng/acts/H-3.3/>

- - Health of Animals Regulations (C.R.C., c. 296)

<https://laws-lois.justice.gc.ca/eng/regulations/C.R.C.,_c._296/>

- List of Security Sensitive Biological Agents (agents having dual-use potential) <https://www.canada.ca/en/public-health/services/laboratory-biosafety-biosecurity/human-pathogens-toxins-act/security-sensitive-biological-agents.html>
- Export and Import Permits Act (EIPA): implements export controls through the Export Control List (ECL)

<https://laws-lois.justice.gc.ca/eng/regulations/sor-89-202/FullText.html>

- - Dual-use list (goods and technology)

<https://www.international.gc.ca/controls-controles/about-a_propos/expor/guide-2016.aspx?lang=eng#group1>

- - Chemical and Biological Weapons Non-Proliferation List

<http://www.international.gc.ca/controls-controles/about-a_propos/expor/guide-2016-2.aspx?lang=eng#group7>

A new guidance document is currently under development: <https://www.canada.ca/en/public-health/programs/consultation-biosafety-guideline-dual-use-life-science-research/document.html>

- 1. GMO Transboundary Movement – Cartagena Protocol Implementation [(home)](#_top)

Canada is not a party to the Cartagena Protocol: <https://www.canada.ca/en/environment-climate-change/corporate/international-affairs/partnerships-organizations/biosafety-cartagena-protocol.html>

Canada supports the environmental objectives of the Biosafety Protocol and signed the agreement in April 2001. However, Canada has not ratified the Protocol because of concerns with its lack of clarity and predictability in terms of its implementation and enforcement.

Canada has a strong regulatory framework for biosafety risk assessment that involves regulating products with novel traits. Living modified organisms are regulated under the Plant Protection Act and the Seeds Act (living modified plants), the Canadian Environmental Protection Act 1999 (living modified animals), the Fisheries Act (for aquatic organisms) and the Health of Animals Act (veterinary biologics).

- 1. Access and Benefit Sharing of Genetic Resources – Nagoya Protocol [(home)](#_top)

Canada is not a party to the Nagoya Protocol:

<https://www.canada.ca/en/environment-climate-change/corporate/international-affairs/partnerships-organizations/nagoya-protocol-access-genetic-resources>

There is currently no single, comprehensive access and benefit-sharing (ABS) system in place in Canada to govern access to genetic resources and associated traditional knowledge or to facilitate the sharing of benefits arising from their use.

Currently, some laws and regulations at the federal, provincial and territorial levels cover some of the elements of ABS for various genetic resources in Canada.

- 1. Plant Health – Import/Export Regulations, Traceability [(home)](#_top)

Plant Protection Act (S.C. 1990, c. 22).

<https://laws-lois.justice.gc.ca/eng/acts/P-14.8/>

Plant Protection Regulations (SOR/95-212).

<https://laws-lois.justice.gc.ca/eng/regulations/SOR-95-212/>

Specific guidance document from Canada for handling plant pests:

<http://www.inspection.gc.ca/plants/plant-pests-invasive-species/biocontainment/containment-standards/eng/1412353866032/1412354048442>

- 1. Animal health – Notifiable Diseases, Emergency Measures [(home)](#_top)

Health of Animals Act (S.C. 1990, c. 21)

<https://laws-lois.justice.gc.ca/eng/acts/H-3.3/>

Health of Animals Regulations (C.R.C., c. 296)

<https://laws-lois.justice.gc.ca/eng/regulations/C.R.C.,_c._296/>

- 1. Occupational Hygiene (*e.g. Legionella*) [(home)](#_top)

Human Pathogen and Toxins Act (S.C. 2009, c. 24)

<https://laws.justice.gc.ca/eng/acts/H-5.67/>

Human Pathogens and Toxins Regulations (SOR/2015-44)

<https://laws.justice.gc.ca/eng/regulations/SOR-2015-44/>

- 1. Transport [(home)](#_top)

Within Canada:

Transportation of Dangerous Goods Act, 1992 (S.C. 1992, c. 34).

Transportation of Dangerous Goods Regulations (SOR/2001-286).

Worldwide:

ADR (road)

IATA (air)

IMDG (sea)

# Australia Status: Sep ’19 [(home)](#_top)

- 1. Worker Protection [(home)](#_top)

Work Health and Safety (WHS) Act

<https://www.legislation.gov.au/Details/C2018C00293>

Work Health and Safety (WHS) Regulations <https://www.legislation.gov.au/Details/F2019C00050>

- 1. GMO Contained Use and Deliberate Release [(home)](#_top)

The Commonwealth Gene Technology legislation consists of the following:

- The Gene Technology Act 2000 (current compilation) <http://www.comlaw.gov.au/Current/C2004C04256>
- The Gene Technology Regulations 2001 (current compilation) <http://www.comlaw.gov.au/Current/F2007C00201>
- The Gene Technology (Consequential Amendments) Act 2000 <http://www.comlaw.gov.au/Current/C2004B00718>
- The Gene Technology (Licence Charges) Act 2000 <http://www.comlaw.gov.au/Current/C2004A00764>

Guidelines for the Transport, Storage and Disposal of GMOs (Version 1.1). <http://www.ogtr.gov.au/internet/ogtr/publishing.nsf/Content/tsd-guidelines-toc>

The website of the Office of Gene Technology Regulator (OGTR) provides practical information on accreditation, responsibilities of accredited organizations and Institutional Biosafety Committees (IBCs), list of accredited organizations, and guidelines and application forms to apply for work with GMOs. It also provides information about National IBC forum. <https://www1.health.gov.au/internet/ogtr/publishing.nsf/Content/section-working-with-gmos>

- 1. Pathogen Work – Plants [(home)](#_top)

Biosecurity Act 2015 <https://www.legislation.gov.au/Details/C2019C00097>

Biosecurity Regulation 2016 <https://www.legislation.gov.au/Details/F2018C00579>

Import conditions apply to live cultures of plant-related microorganisms and plant material infected with live microorganisms (including plant pathogens and symbionts), such as viruses, viroids, fungi, stramenopiles and bacteria. Prior to the importation into Australian territory, a valid import permit issued by the Department of Agriculture is required. The conditions for importation will be determined on a case by case assessment. These types of goods generally require Biosecurity Containment in a BC2 or BC3 Approved Arrangement site. The permit assessment will also determine conditions such as those relating to on-arrival inspections and the storage, use, movement, transfer and release of the goods.

- 1. Pathogen Work – Animals [(home)](#_top)

Pure cultures, or primary derivatives (e.g. antigens, proteins, genetic material), of a standard laboratory microorganism or infectious agent for *in-vitro* or *in-vivo* use in laboratory organisms only, do not require biosecurity containment (standard laboratory microorganisms and infectious agents), because they are endemic (occur in Australia) and are commonly imported by laboratories in Australia. <https://bicon.agriculture.gov.au/BiconWeb4.0/ViewElement/Element/Index?elementPk=1070811&caseElementPk=1132020>

For any other micro-organisms and infectious agents, a valid import permit issued by the Department of Agriculture is required. The department will assess the application and based on that assessment may decide to grant an import permit subject to any conditions deemed necessary for safe importation, use and disposal of those products. Note: Some microorganisms and infectious agents require mandatory biosecurity containment in an approved arrangement (AA) site. This will be determined on assessment of the application.

- 1. Waste [(home)](#_top)

Covered by the different topic-specific legislative texts.

- 1. Dual-Use Items / DURC / Biosecurity [(home)](#_top)

Customs Act 1901

<http://www.legislation.gov.au/Details/C2013C00381>

Customs (Prohibited Exports) Regulations 1958*,* **Regulation 13E** <http://www.legislation.gov.au/Series/F1996B03403>

The Defense and Strategic Goods List – Part 2 Dual Use List - Category 1 - Materials, Chemical, Micro-organisms and Toxins.

<https://dsgl.defence.gov.au/Pages/Home.aspx>

Crimes (Biological Weapons) Act 1976 <https://www.legislation.gov.au/Details/C2018C00180>

Crimes (Biological Weapons) Regulations 2019 <https://www.legislation.gov.au/Details/F2019L00468>

- 1. GMO Transboundary Movement – Cartagena Protocol Implementation [(home)](#_top)

Transboundary movement of GMOs is covered by Australia's National Regulatory Scheme for GMOs:

- The Gene Technology Act 2000 (current compilation) <http://www.comlaw.gov.au/Current/C2004C04256>
- The Gene Technology Regulations 2001 (current compilation) <http://www.comlaw.gov.au/Current/F2007C00201>
- The Gene Technology (Consequential Amendments) Act 2000 <http://www.comlaw.gov.au/Current/C2004B00718>
- The Gene Technology (Licence Charges) Act 2000 <http://www.comlaw.gov.au/Current/C2004A00764>

Guidelines for the Transport, Storage and Disposal of GMOs (Version 1.1). <http://www.ogtr.gov.au/internet/ogtr/publishing.nsf/Content/tsd-guidelines-toc>

- 1. Access and Benefit Sharing of Genetic Resources – Nagoya Protocol [(home)](#_top)

Australia signed the Protocol in January 2012 and is now developing its approach to implementation and ratification. Australia’s existing domestic measures are consistent with the Protocol, but it needs to develop measures to make sure that the genetic resources and associated traditional knowledge used in Australia were legally acquired in the country from which they come. Australia can only ratify the Protocol when it is confident that all obligations are being met – this requires changes to domestic law.

Australia’s existing domestic measures for access to Biological resources:

Each State or Territory government manages access to biological resources in its jurisdiction under its own laws, in line with the endorsement of the Nationally consistent approach for access to and the utilization of Australia's native genetic and biochemical resources. <http://www.environment.gov.au/biodiversity/publications/access/nca/index.html>

If you wish to obtain biological resources from a Commonwealth area for the purpose of research and development on any genetic resources, or biochemical compounds, comprising or contained in the biological resources, you will need to obtain a permit under Part 8A of the Environment Protection and Biodiversity Conservation Regulations 2000. <http://www.environment.gov.au/biodiversity/science/access/commonwealth/index.html>

<https://www.legislation.gov.au/Details/F2018C00929>

If the location of your proposed research is not in a Commonwealth area, you should contact relevant State and Territory Authorities. <http://www.environment.gov.au/topics/science-and-research/australias-biological-resources/access-biological-resources-states-and>

- 1. Plant Health – Import/Export regulations, Traceability [(home)](#_top)

The National Plant Health Committee (PHC)​​​​​​ is the peak government plant biosecurity policy and decision-making forum. More information can be found on the website of the department of Agriculture: <http://www.agriculture.gov.au/>.

Biosecurity Act 2015 <https://www.legislation.gov.au/Details/C2019C00097>

Biosecurity Regulation 2016 <https://www.legislation.gov.au/Details/F2018C00579>

National priority plant pests 2016 (exotic to Australia, under eradication or have limited distribution). <http://www.agriculture.gov.au/pests-diseases-weeds/plant/national-priority-plant-pests-2016>

Import conditions for specific materials can be consulted at the Biosecurity Import Conditions system (BICON). <https://bicon.agriculture.gov.au/BiconWeb4.0>

Plant health aspects of transport within Australia are managed by the different states and territories.

More information can be found on the websites of the lead agencies in the states and territories:

- Australian Capital Territory: Environment Planning and Sustainable Development Directorate (EPSD) <http://environment.act.gov.au>
  - Plant Diseases Act 2002
  - Pest Plants and Animals Act 2005
- New South Wales: Department of Primary Industries (NSW DPI) [http://www.dpi.nsw.gov.au](http://www.dpi.nsw.gov.au/)
  - NSW Biosecurity Act 2015
- Northern Territory: NT Department of Primary Industry and Resources (NT DPIR) <http://dpir.nt.gov.au>
  - Plant Health Act 2008
  - Plant Health Regulations 2011
- Queensland: Queensland Department of Agriculture and Fisheries (QDAF) <http://www.daf.qld.gov.au>
  - Biosecurity Act 2014
  - Biosecurity Regulation 2016
- South Australia: Department of Primary Industries and Regions SA (PIRSA) <http://www.pir.sa.gov.au>
  - Health Act 2009
  - Plant Health Regulations 2009
- Tasmania: Department of Primary Industries, Parks, Water and Environment (DPIPWE) <http://www.dpipwe.tas.gov.au>
  - Plant Quarantine Act 1997
- Victoria: Victorian Department of Jobs, Precincts and Regions (DJPR Vic) <http://djpr.vic.gov.au>
  - Plant Biosecurity Act 2010
- Western Australia: Department of Primary Industries and Regional Development (DPIRD) <http://dpird.wa.gov.au>
  - Biosecurity and Agriculture Management Act 2007

- 1. Animal Health – Notifiable Diseases, Emergency Measures [(home)](#_top)

Animal pests and diseases that must be reported:

- National List of Notifiable Animal Diseases

<http://www.agriculture.gov.au/pests-diseases-weeds/animal/notifiable>

- National List of Reportable Diseases of Aquatic Animals

<http://www.agriculture.gov.au/animal/aquatic/reporting>

- State and Territory Notifiable Animal Diseases lists

<http://www.agriculture.gov.au/pests-diseases-weeds/animal/state-notifiable>

Biosecurity Act 2015 <https://www.legislation.gov.au/Details/C2019C00097>

Biosecurity Regulation 2016 <https://www.legislation.gov.au/Details/F2018C00579>

Import conditions for specific materials can be consulted at the Biosecurity Import Conditions system (BICON). <https://bicon.agriculture.gov.au/BiconWeb4.0>

- 1. Occupational Hygiene (*e.g. Legionella*) [(home)](#_top)

Guidelines for Legionella Control (2016) <https://www1.health.gov.au/internet/main/publishing.nsf/Content/A12B57E41EC9F326CA257BF0001F9E7D/$File/Guidelines-Legionella-control.pdf>

Facility Managers should consult relevant state or territory legislation and guidelines for the management and control of *Legionella* in cooling towers.

- 1. Transport [(home)](#_top)

ADR (road)

IATA (air)

IMDG (sea)

# Singapore Status: Aug ’19 [(home)](#_top)

- 1. Worker protection [(home)](#_top)

Workplace Safety and Health Act

<https://sso.agc.gov.sg/Act/WSHA2006>

- 1. GMO Contained Use and Deliberate Release [(home)](#_top)

Singapore Biosafety Guidelines for Research on GMOs (the “GMAC Research Guidelines”) <https://www.gmac.sg/pdf/Research/Singapore%20Biosafety%20Guidelines%20for%20GMO%20Research_Jan%202013.pdf>

Singapore Guidelines on the Release of Agriculture-Related GMOs (the “GMAC Release Guidelines”)

<https://www.gmac.sg/pdf/Agriculture_Guidelines.pdf>

Multi-Agency Joint Circular on Guidelines for Research, Release, and Importation of GMO'sopens in a new window

<https://www.moh.gov.sg/docs/librariesprovider7/useful-info-and-guidelines-documents/multi-agency_circular_on_biosafety_guidelines_for_gmos_final_7_nov_2008.pdf?sfvrsn=f0e8f3dc_2>

More information is available on the GMAC website: <https://www.gmac.sg/Index_Guidelines_Overview_on_GMAC_Guidelines.html>

- 1. Pathogen Work – Plants [(home)](#_top)

No information available

- 1. Pathogen Work – Animals [(home)](#_top)

Biological Agents and Toxins Act and associated guidance documents:

- National Biosafety Standards for Maximum Containment Facilitiesopens in a new window <https://www.moh.gov.sg/docs/librariesprovider7/useful-info-and-guidelines-documents/national-biosafety-standards-for-mcf-may-2019.pdf>
- Risk Assessment Guidelinesopens in a new window <https://www.moh.gov.sg/docs/librariesprovider7/useful-info-and-guidelines-documents/guideline-on-laboratory-activity-risk-assessment-finaljul11.pdf?sfvrsn=be9a5fc8_2>
- Guidelines on the Import and Transshipment of Biological Agents and Toxinsopens in a new window <https://www.moh.gov.sg/docs/librariesprovider7/useful-info-and-guidelines-documents/guidelines_on_the_import_and_transhipment_of_ba_and_toxins.pdf?sfvrsn=f1dc64f0_2>
  1. Waste [(home)](#_top)

Hazardous Waste (Control of Export, Import and Transit) Act <https://sso.agc.gov.sg/Act/HWCEITA1997>

Hazardous Waste (Control of Export, Import and Transit) Regulations <https://sso.agc.gov.sg/SL/122A-RG1>

- 1. Dual-Use Items / DURC / Biosecurity [(home)](#_top)

Security Guidelines for Premises Storing or Handling Security Sensitive Materialsopens in a new window <https://www.moh.gov.sg/docs/librariesprovider7/useful-info-and-guidelines-documents/ssm-pswg-guidelines-(final-draft)(2)-1.pdf?sfvrsn=34107734_2>

Circular on Physical Security for Facilities Handling First Schedule Part I, Third Schedule and Fourth Schedule biological agents under the Biological Agents and Toxins Actopens in a new window <https://www.moh.gov.sg/docs/librariesprovider7/useful-info-and-guidelines-documents/circular-on-physical-security-for-facilities-handling-bat-(moh-mha)-(3)-(2).pdf?sfvrsn=3978cc8d_2>

- 1. GMO Transboundary Movement – Cartagena Protocol Implementation [(home)](#_top)

Singapore is not a party to the Cartagena Protocol: <https://www.cbd.int/information/parties.shtml#tab=1>

Common ASEAN framework for assessment of risks associated with the transboundary movement of agriculture-related GMOs: [ASEAN Guidelines on Risk Assessment of Agriculture-Related Genetically Modified Organisms (GMOs)](http://asean.org/?static_post=asean-guidelines-on-risk-assessment-of-agriculture-related-genetically-modified-organisms-gmos)

- 1. Access and Benefit Sharing of Genetic resources – Nagoya Protocol [(home)](#_top)

Singapore is not a party to the Nagoya Protocol: <https://www.cbd.int/information/parties.shtml#tab=2>

- 1. Plant Health – Import/Export Regulations, Traceability [(home)](#_top)

Control of Plants Act (<https://sso.agc.gov.sg/Act/CPA1993>) and associated rules for specific topics:

- Control of Plants (Phytosanitary Certification) Rules <https://sso.agc.gov.sg/SL/57A-R2>
- Control of Plants (Import and Transhipment of Fresh Fruits and Vegetables) Rules <https://sso.agc.gov.sg/SL/57A-R1>
- Control of Plants (Plant Importation) Rules <https://sso.agc.gov.sg/SL/57A-R4>
- Control of Plants (Cultivation of Plants) (Licensing and Certification) Rules <https://sso.agc.gov.sg/SL/57A-R2>
  1. Animal health – Notifiable Diseases, Emergency Measures [(home)](#_top)

Animals and Birds Act (<https://sso.agc.gov.sg/Act/ABA1965>) and associated rules for specific topics:

- Animals and Birds (Importation) Order 2009

<https://sso.agc.gov.sg/SL/ABA1965-S126-2009>

- Animals and Birds (Disease) Notification <https://sso.agc.gov.sg/SL/7-N2>
- Animals and Birds (Pigeons) Rules <https://sso.agc.gov.sg/SL/7-R4>
- Animals and Birds (Care and Use of Animals for Scientific Purposes) Rules <https://sso.agc.gov.sg/SL/7-R10>

Infectious Diseases Act <https://sso.agc.gov.sg/Act/IDA1976>

- Infectious Diseases (Quarantine) Regulations

<https://sso.agc.gov.sg/SL/137-RG1>

- Infectious Diseases (Measures to Prevent Spread of Infectious Diseases) (No. 2) Order 2007 <https://sso.agc.gov.sg/SL/IDA1976-S75-2007>
- Infectious Diseases (Notification of Prescribed Infectious Diseases) Regulations 2008 <https://sso.agc.gov.sg/SL/IDA1976-S612-2008>
  1. Occupational Hygiene (*e.g. Legionella*) [(home)](#_top)

No information available

- 1. Transport [(home)](#_top)

ADR (road)

IATA (air)

IMDG (sea)

# Brazil Status: Nov ’19 [(home)](#_top)

- 1. Worker Protection [(home)](#_top)

Decreto Lei 5452/1943: Consolidação das Leis do Trabalho – CLT

(establishes worker protection rules)

<http://www.planalto.gov.br/ccivil_03/decreto-lei/Del5452compilado.htm>

References to norms relevant to Biosafety:

- NR-04: Norma regulamentadora do Ministério do Trabalho nº 4 - Serviços Especializados em Engenharia e de Segurança e em Medicina do Trabalho

(How SESMT* should work - *specialized services in safety engineering and worker health and welfare = HSE)

- NR-05: Norma Regulamentadora do Ministério do Trabalho nº 5 - CIPA - Comissão Interna de Prevenção de Acidentes

(When and how to establish a CIPA, that should identify, prevent and deal with work hazards and accidents)

- NR-06: Norma Regulamentadora do Ministério do Trabalho nº 6 - EPI - Equipamentos de Proteção Individual

(What are the personal protective equipment (PPE) and their characteristics/ technical specifications for workers health and welfare protection/assurance)

- NR-07: Norma Regulamentadora do Ministério do Trabalho nº 7 - PCMSO - Programa de Controle Médico de Saúde Ocupacional

(Minimal requirements for Medical check-ups for occupational health, depending on company economic activity and number of permanent employees)

- NR-09: Norma regulamentadora do Ministério do Trabalho nº 9 - PPRA - Programa de Prevenção de Riscos Ambientais

(Mandatory rules for environmental protection and workers' health and integrity protection)

- NR-15: Norma regulamentadora do Ministério do Trabalho nº 15 - Atividades e Operações Insalubres

(How to manage unhealthy (insalubrious) activities - establish tolerance limits for insalubrious operations)

- NR-16: Norma regulamentadora do Ministério do Trabalho nº 16 - Atividades e operações perigosas

(Lists the operations/activities considered dangerous)

- NR-24: Norma regulamentadora do Ministério do Trabalho nº 24 - Condições sanitárias e de conforto no local de trabalho

(Determines the minimum conditions of health according to the type of work and staff number)

- NR-31: Norma regulamentadora do Ministério do Trabalho nº 31 - Segurança e Saúde no Trabalho na Agricultura, Pecuária, Silvicultura, Exploração Florestal e Aquicultura

(Rules on the safety and health of workers in agricultural and extractive activity)

- NR-32: Norma regulamentadora do Ministério do Trabalho nº 32 - Segurança e Saúde no Trabalho em Serviços de Saúde (Riscos Biológicos)

(Establishes guidelines for the implementation of measures to protect the safety and health of people working in contact with biological fluids and biological and hospital waste)

- 1. GMO Contained Use and Deliberate Release [(home)](#_top)

Lei 11.105/2005: Lei de Biosegurança (Brazilian Biosafety Law) <https://www.planalto.gov.br/ccivil_03/_Ato2004-2006/2005/Lei/L11105.htm>

Decreto 5.591/2005: Regulamenta os dispositivos da Lei de Biossegurança (implementation of Brazilian Biosafety law) <https://www.planalto.gov.br/ccivil_03/_Ato2004-2006/2005/Decreto/D5591.htm>

GMO Contained use:

- RN-01: Dispõe sobre a instalação e o funcionamento das Comissões Internas de Biossegurança (CIBios) e sobre os critérios e procedimentos para requerimento, emissão, revisão, extensão, suspensão e cancelamento do Certificado de Qualidade em Biossegurança (CQB).

(rules to receive GMO permit, and establish the internal Biosafety Committee) – updated by:

- - RN-11: Altera e atualiza a RN01 - O inciso V e as alíneas "a" a "c" do art. 16 da Resolução Normativa nº 1
  - RN-14: Altera e atualiza a RN01 - O inciso IV do Art. 5°, o caput do art. 9° e os incisos II, IV e VI do art. 11 da Resolução Normativa n° 1
- RN-02: Dispõe sobre a classificação de risco de OGM e os níveis de biossegurança a serem aplicados nas atividades e projetos em contenção com OGM e seus derivados.

(GMO risk assessment and biosafety levels to be applied in relation to activities with GMOs and their derivatives).

GMO Deliberate release:

- RN-03: Monitoramento pós-liberação comercial de milho GM

(GM maize commercial post-release monitoring)

- RN-04: Estabelecer as distâncias mínimas de isolamento a serem observadas entre cultivos comerciais de milho geneticamente modificado e cultivos de milho não geneticamente modificado

(Provisions on minimum isolation distances to be observed between commercial GM maize crops and non-GM maize crops)

- RN-05: Dispõe sobre a liberação comercial de Organismos Geneticamente Modificados - OGM e seus derivados

(Provisions on commercial release of GMOs and their derivatives)

- RN-06: Dispões sobre as liberações planejadas no meio ambiente de Organismos Geneticamente Modificados de origem vegetal e seus derivados

(Provisions on planned releases into the environment of genetically modified organisms of plant origin and their derivatives)

- RN-07: Dispõe sobre as liberações planejadas no meio ambiente de Microorganismos e Animais Geneticamente Modificados de Classe de Risco I e seus derivados

(Provisions on planned releases into the environment of Hazard Class I GM Microorganisms and Animals and their derivatives)

- RN-08: Dispões sobre as liberações planejadas no meio ambiente de Organismos Geneticamente Modificados de origem vegetal e seus derivados que já tenham sido aprovados anteriormente

(Provisions on planned releases into the environment of GM organisms of plant origin and their derivatives that have already been approved)

- RN-09: O monitoramento pós-liberação comercial de Organismos Geneticamente Modificados – OGM ou sua isenção

(Post-commercial release monitoring of GMOs or their exemption)

- RN-15: Liberação comercial de Organismo Geneticamente Modificado – OGM que contenha mais de um evento

(Commercial release GMO containing more than one event)

- Lei 11.460/2007: Dispõe sobre o plantio de organismos geneticamente modificados em unidades de conservação

(Provisions on the planting of GMOs in protected areas) <http://www.planalto.gov.br/ccivil_03/_Ato2007-2010/2007/Lei/L11460.htm>

- 1. Pathogen Work – Plants [(home)](#_top)

The legislation is currently under revision – references to legislation include: IN52/2007, IN41/2008, IN53/2013, IN28/2016, IN29/2016, IN33/2016

- 1. Pathogen Work – Animals [(home)](#_top)

No specific legislation identified

- 1. Waste [(home)](#_top)

No specific legislation identified

- 1. Dual-Use Items / DURC / Biosecurity [(home)](#_top)

No specific legislation identified

- 1. GMO Transboundary Movement – Cartagena Protocol Implementation [(home)](#_top)

Lei 11.105/2005: Lei de Biosegurança (Brazilian Biosafety Law) <https://www.planalto.gov.br/ccivil_03/_Ato2004-2006/2005/Lei/L11105.htm>

Decreto 5.591/2005: Regulamenta os dispositivos da Lei de Biossegurança (implementation of Brazilian Biosafety law) <https://www.planalto.gov.br/ccivil_03/_Ato2004-2006/2005/Decreto/D5591.htm>

Decreto 6.925/2009 Autoridade Nacional Competente

(National Competent Authority)

<http://www.planalto.gov.br/ccivil_03/_Ato2007-2010/2009/Decreto/D6925.htm>

- 1. Access and Benefit Sharing of Genetic Resources – Nagoya Protocol Implementation [(home)](#_top)

No specific legislation identified

- 1. Plant Health – Import/Export Regulations, Traceability [(home)](#_top)

The legislation is currently under revision – references to legislation include: IN52/2007, IN41/2008, IN53/2013, IN28/2016, IN29/2016, IN33/2016

- 1. Animal Health – Notifiable Diseases, Emergency Measures [(home)](#_top)

No specific legislation identified

- 1. Occupational Hygiene (*e.g. Legionella*) [(home)](#_top)

See ‘Worker protection’

- 1. Transport [(home)](#_top)

ADR (road)

IATA (air)

IMDG (sea)
